# The effects of person-centred active rehabilitation on symptoms of suspected Chronic Traumatic Encephalopathy: A mixed-methods single case design

**DOI:** 10.1371/journal.pone.0302260

**Published:** 2024-05-30

**Authors:** Rachael Hearn, James Selfe, Maria I. Cordero, Nick Dobbin

**Affiliations:** 1 Department of Health Professions, Faculty of Health and Education, Manchester Metropolitan University, Manchester, United Kingdom; 2 Department of Psychology, Faculty of Health and Education, Manchester Metropolitan University, Manchester, United Kingdom; University of Catania, ITALY

## Abstract

**Objective:**

The objective was to investigate the effectiveness of a person-centred active rehabilitation programme on symptoms associated with suspected Chronic Traumatic Encephalopathy (CTE). This was accomplished by (1) assessing the effect that a person-centred active rehabilitation programme had on participant symptoms, and (2) exploring how temporal contextual factors affected the participants’ experience with, and perceived effectiveness of, the active rehabilitation programme.

**Methods:**

A twelve-month mixed-methods single case experimental research design was used with six cases (participants). Individual cases were involved in a 51-week study period including an initial interview and three-week baseline phase. Cases were then randomly allocated to one of two n-of-1 study designs (i.e., A-B, B-A, B-A, A-B or B-A, A-B, A-B, B-A) where A and B represent a non-intervention and intervention phase, respectively. Interviews were conducted regularly throughout the study whilst outcome measures were assessed at each follow-up. Analysis of the data included visual, statistical, and qualitative analysis.

**Results:**

Visual and statistical analysis of cognitive and executive function, and mindful attention, demonstrated trivial-to-large effects with the summary reflecting positive or unclear results. A mixed picture was observed for mood and behaviour with effects considered trivial-to-large, and the summary demonstrating positive, unclear and negative effects. Qualitative analysis indicated a perceived improvement in outcome measures such as memory, attention, anxiety, and emotional control despite mixed quantitative findings whilst a clear impact of contextual factors, such as COVID-19, the political atmosphere, exercise tolerance, programme progression, and motivation were evident during the intervention.

**Conclusions:**

This study has provided primary-level evidence to suggest active rehabilitation as a potential intervention for the management of suspected CTE symptoms. This study has also demonstrated the benefit of a person-centred approach to both clinical research and practice, particularly by considering contextual factors for a better understanding of an intervention effect.

## Introduction

Chronic Traumatic Encephalopathy (CTE) was formally defined by the National Institute of Neurological Disorders and Stroke/National Institute of Biomedical Imaging and Bioengineering (NINDS/NIBIB) consensus panel in 2015. CTE is a neurodegenerative pathology defined by its unique, irregular pattern of tau protein accumulation around small blood vessels at the base of the cortical sulci [1]. While CTE has been known by many since at least 1928, the clinical profile has consistently been linked to an exposure to repetitive brain injury. CTE has now been diagnosed post-mortem in former American football, football (soccer), rugby, Australian rules football, ice hockey, baseball, and wrestling athletes, as well as military personnel and domestic abuse victims [1–3]. The inability to diagnose CTE pre-death has led to the development of a clinical profile termed Traumatic Encephalopathy Syndrome (TES). This clinical profile has enabled researchers to identify individuals with symptoms associated with suspected CTE allowing for early management and development of active rehabilitation or treatment options [2, 4–6].

Currently, no evidence-based therapy has been developed to treat or manage symptoms associated with CTE to our knowledge. Cantu and Budson [4] provided the first expert review outlining potential lifestyle modifications and symptom management strategies for CTE. Recommendations included exercise, active rehabilitation, diet, cognitive rehabilitation, mood/behavioural therapy, occupational therapy, vestibular and motor therapy, and pharmacological therapy. This expert opinion has been reiterated by Fusco et al. [7] and Rossi et al. [8] in expert opinions or narrative reviews. Themes including cognitive and motor rehabilitation therapy, mindfulness, mood/behavioural therapy, occupational therapy, diet, exercise, and active rehabilitation were suggested to help manage neuropsychiatric symptoms of CTE [7, 8]. Finally, an umbrella review [9] has reported the effect that active rehabilitation has on other tauopathies with symptoms associated with CTE. This review found that various forms of active rehabilitation had a positive effect (standardised mean difference ranging from 0.11 to 0.88) on symptoms of cognitive and motor function in populations diagnosed with Alzheimer’s and Parkinson’s disease.

Despite being an area of growing interest amongst researchers and clinicians, current evidence is largely limited to secondary level research. Whilst various forms of management strategies have been suggested (e.g., active rehabilitation), few have examined the efficacy of this. It is also important to consider the patient within a rehabilitation or management approach. A person-centred care approach allows for the management of patients with a unique set of symptoms which is likely to be particularly important when managing individuals with suspected CTE. Accordingly, the objective of this study was to investigate the effectiveness of person-centred active rehabilitation on symptoms of TES, providing the first primary level research exploring a potential management for CTE symptoms. This was accomplished by: (1) assessing the effect that a person-centred active rehabilitation programme had on participant symptoms suspected to be associated with the development of CTE, and (2) exploring how proximal (individual—interpersonal and intrapersonal) and distal (environmental—socio-economic, rural-urban differences and immigration background) factors affected the participants’ experience with, and perceived effectiveness of, the active rehabilitation programme through repeated interviews during the course of the intervention.

## Materials and methods

A mixed-methods single case research (MMSCR) design was used for this study with an n-of-1 framework. The study was designed and reported in line with the Consolidated Standards of Reporting Trials (CONSORT) extension for N-of-1 trials [10, 11]. Ethical approval was granted by the Faculty of Health, Psychology and Social Care Research Ethics and Governance Committee at Manchester Metropolitan University (ID: 11822).

### Eligibility criteria

Individuals between the age 20 and 60 years who met the 2014 TES criteria [6], spoke/read English, and were at least one year retired from competitive sport were eligible for inclusion. The 2014 TES criteria was used as the updated criteria by Katz and colleagues [5] was not available at the start of the study. An age range of 20 to 60 years allowed for sufficient exposure to mTBI/contact sport while minimising the chance of concomitant neurodegenerative disease, presence of dementia, and other neurological disorders. Individuals diagnosed with dementia were excluded.

At any time during data collection, withdrawal could be explicitly expressed by the participant. Withdrawal was assumed if the participant (1) missed more than two follow-up interviews in a row, or (2) did not respond to at least two contact attempts made by the researcher seeking to schedule a follow-up interview. Withdrawal was also discussed if the participant expressed dissent with the study procedures. In the event of participant withdrawal, any data where a full data collection cycle had been completed (A-B matched pair) was included in the analysis.

### Materials and procedures

#### General procedure

**Fig 1:**
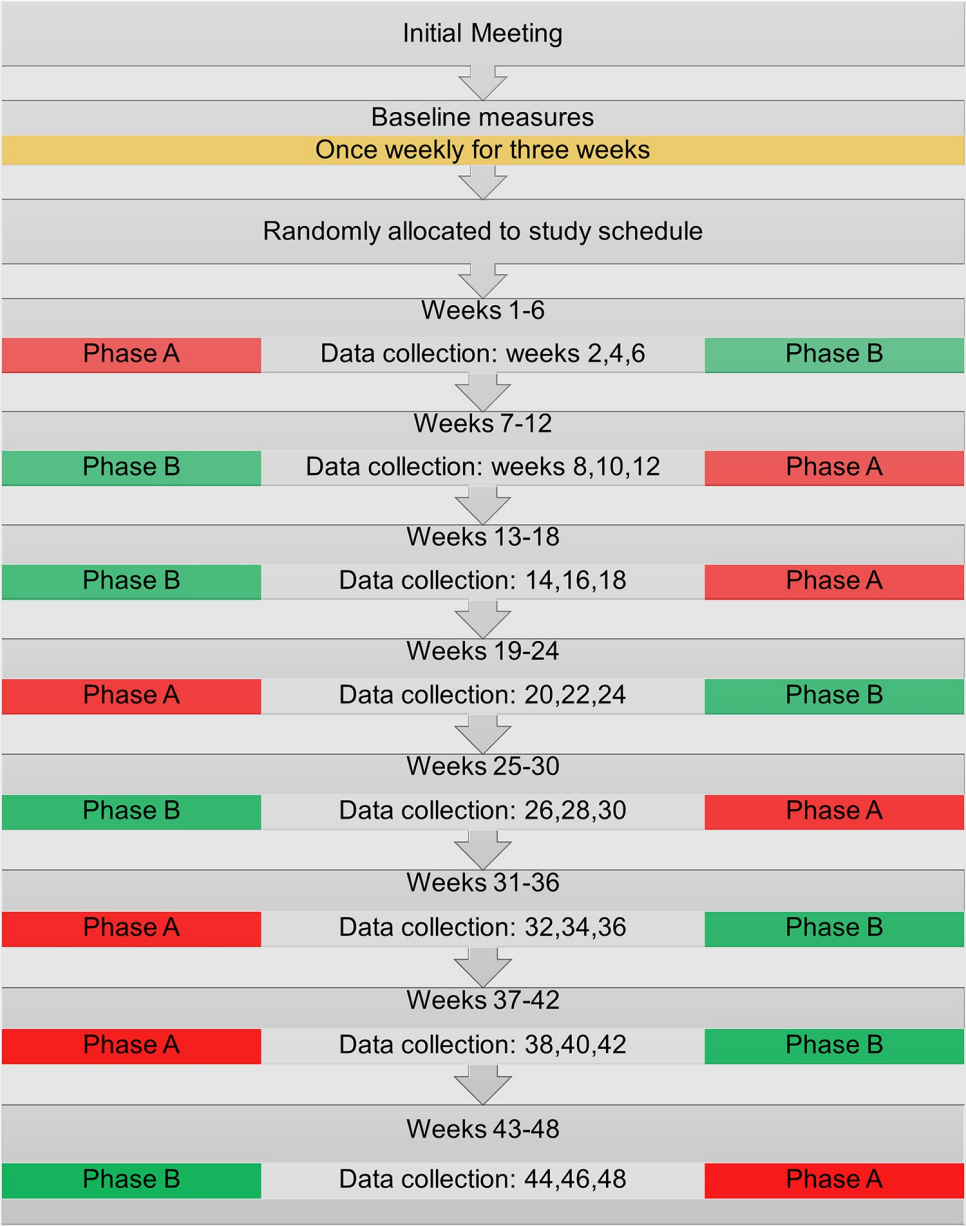
Schematic overview of the data collection period. Note: A = non-intervention phase. B–intervention phase.

Individual cases (participants) were involved for 51 weeks, with recruitment and study commencement occurring on a rolling basis starting in April 2020 and ending in June 2021. The study began with an initial interview, followed by a three-week baseline phase. Participants were then randomly allocated to one of two systematic counterbalanced n-of-1 study designs, the first being A-B, B-A, B-A, A-B and the second being B-A, A-B, A-B, B-A, where ‘A’ indicates a non-intervention phase and ‘B’ indicates an intervention phase. Each paired phase (A-B or B-A) lasted twelve weeks and consisted of six interviews which took place every two weeks. A schematic overview of the study is illustrated in Fig 1.

#### Initial interview and screening assessments

An initial interview was conducted to (1), screen the participant for study eligibility, ensuring they met clinical criteria for the presence of TES, and (2), to screen the participant for evidence of potential cognitive impairment, changes in mood/behaviour, or motor impairment associated with CTE which could be assessed during the three-week baseline period. To date, there is no established battery of assessments relevant to the population used in this study; thus, a pragmatic approach was adopted. The initial interview began with three core screening assessments to measure levels of cognitive function, mood/behavioural symptoms, and motor function (S1 Table). The initial meeting concluded with a semi-structured interview that sought to elicit further information related to the eligibility criteria and provided the participant with an opportunity to share information on relevant sporting history, medical history, family medical history, and any other further symptoms or concerns. In line with a person-centred care approach, participants also gave information regarding activity capabilities and preferences to inform the design of the active rehabilitation programme.

#### Baseline phase and follow-ups

The three-week baseline period sought to establish the presence of measurable impairment. The assessments used were individualised for each case based on relevance and symptoms reported in the initial interview. Any symptom of TES had the potential for inclusion. A description of all assessments used in the study can be found in S2 Table. To reduce the burden on participants, a five-assessment limit was implemented. For those who did not reach the five-assessment limit, the Perceived Stress Scale (PSS) was included to provide further support to the contextual information gathered during the follow-up interviews.

During each follow-up, participants completed an online survey consisting of self-report symptom assessments. The online survey also gave open box to submit a daily activity log. This log allowed participants to report daily log of their physical activity as well as additional contextual information related to their symptoms and programme experience. Participants also took part in a semi-structured follow-up interview at each data collection point. The semi-structured interviews primarily sought to create a person-centred care environment, promoting factors such as understanding the participant as a person and encouraging involvement in the co-design of the active rehabilitation. The aim of these interviews was to understand i) how the presence of the person-centred active rehabilitation programme affected the symptoms of interest, ii) how the participant described their experience with the rehabilitation programme and prescription, iii) how proximal and distal factors may have influenced the participants symptom levels, and iv) how proximal and distal factors may have influenced their experience with or effect of the programme.

#### Intervention delivery

The setting of the study was entirely online. Any outcome assessments used were recorded via Qualtrics XM (Qualtrics, Provo, Utah, USA). The intervention programme was distributed via email and accompanied with online tutorial videos.

During an intervention phase (B phase), participants completed one resistance training session and one cardiovascular session each week. During a non-intervention phase (A phase) the prescribed exercises were removed, but participants were allowed to continue with habitual activities. Care was taken to ensure programme prescriptions resulted in a greater training load during intervention phases relative to the non-intervention phases.

The training programme was tailored by mode, duration, and intensity based on participant needs, preferences, facilities, and strength/fitness levels as understood from the semi-structured interview. Intensity for the resistance training programmes was prescribed using a modified rating of perceived exertion (RPE) scale [12]. The intensity of the cardiovascular training programme was prescribed using the Borg 6–20 scale given its linear relationship with heart rate [12].

### Data analysis

#### Quantitative analysis

Visual analysis of the quantitative data followed a modified framework presented by Wolfe and colleagues [13], created in accordance with The What Works Clearinghouse (WWC) Single-Case Design Standards [14]. Initially, a trend in the data was established for each of the paired non-intervention and intervention phases using a split middle trend (SMT) line [15]. The SMT line was then used to predict outcome measures for the subsequent phase. The size of the effect was estimated using a modified point system [13] based on the following:

- Evidence of change in level, trend and/or variability = 1.0 point
- Change was immediate, there was less than 30% of data overlap, or there was evidence of consistency between phase-types (intervention/non-intervention) = 0.25 point

After summing the scores, 0–2 indicates unclear behavioural change; 3–4 a small behavioural change; 5–6 a moderate behavioural change; 7–8 a large behavioural change.

To support the visual analysis and future research (e.g., sample size, meta-analyses), within-case standardized mean difference (WC-SMD) and non-overlap of all pairs (NAP) with 95% confidence limits were calculated [16]. SMDs were classified as: <2.0 trivial; 0.20–0.50, small; 0.51–0.80, moderate; >0.80, large [17]. NAP was interpreted as a probability that a randomly selected datapoint in the intervention (B) phase was above or below (depending on if an increase or decrease is desirable) a randomly selected datapoint in the non-intervention (A) phase [16].

#### Qualitative analysis

All interviews were transcribed *verbatim*. Transcriptions were then read to identify content related to the topics of interest based on deductive analysis *a-priori*. Additional points of interest that also emerged were included via inductive analysis. To maintain trustworthiness through confirmability, all authors were given a random selection of transcripts to analyse. Qualitative analysis followed the explanation building approach outlined by Yin [18]. The following propositions were initially determined:

1. The presence of the person-centred active rehabilitation programme had a positive effect on the participant’s symptoms of interest.
2. The needs and preferences of the participant regarding the rehabilitation mode and prescription were met.
3. Proximal and distal factors influenced i) the participant’s reported symptom levels, ii) the participant’s experience with the active rehabilitation programme, and iii) the effect of the active rehabilitation programme.

While a total of twenty-four follow-up interviews were available, a pragmatic approach for qualitative analysis was adopted. The sample of interviews used in this study consisted of those that took place at the end of each A-B paired phase (e.g., A1.3, B2.3, B3.3, and A4.3) to provide a more global view of the participant perspective as it related to the phase (whether A or B) and proximal and distal factors. A summary of results is presented in a narrative and visual format.

## Results

### Participant characteristics

Ten participants were recruited for the study. Two participants did not demonstrate a measurable impairment during the screening or baseline phase in one of the three core clinical features and therefore did not meet eligibility criteria. A further two participants withdrew from the study before they completed an entire A-B phase. In both instances, the participant did not respond to at least two contact attempts made by the primary author to schedule follow-up interviews. A reason for withdrawal was not stated by either participant. In line with the study protocol, their data was withdrawn from the study. A total of six participants completed all aspects and were included in the final analysis.

Participant characteristics, informed by the initial semi-structured interview and baseline phase, can be found in Table 1. Results are presented using pseudonyms to maintain anonymity.

**Table 1:** Participant characteristics.

	Niall	Luigi	Kristen	Abel	Gemma	Simon
Age (years)	20	38	29	24	29	35
Sex	Male	Male	Female	Male	Female	Male
Nationality	UK	Canada	USA	USA	USA-Italy	UK
History of head impacts	> 6 years participation in field hockey, basketball, rugby	> 6 years participation in ice hockey; > 4 diagnosed concussion; multiple concussions in short timeline	> 6 years participation in rugby; > 4 diagnosed concussion; multiple concussions in short timelines; 2 episodes of moderate TBI	> 6 years participation in American football, football, rugby	> 6 years participation in karate, basketball, softball, volleyball	> 6 years participation in rugby; 1 episode of moderate TBI
Screening						
*SLUMS*	27/30 AU–WNL	25/30 AU—MCI	30/30 AU—WNL	27/30 AU—WNL	30/30 AU—WNL	23/30 AU—MCI
*GMHAT*	Concentration, worry, anxiety, loss of interest	Concentration, worry, anxiety, depression, loss of interest	Concentration, worry, anxiety, depression, loss of interest, hopelessness, sleep disruption	Concentration, worry, anxiety, depression, loss of interest	Concentration, worry, anxiety,	Concentration, worry, anxiety, depression
*PROMIS*	100/102 AU–normal	100/102 AU–normal	98/102 AU–Mild signs of impairment, largely because of recent surgery	101/102 AU–normal	102/102 AU—normal	100/102—normal
Additional history	Memory impairment, executive dysfunction, history of depression, suicidal tendencies, social isolation	Memory impairment, concentration and attention impairment, executive dysfunction, irritability, short fuse, explosivity, history of panic attack, history of suicidal ideation, persistent headaches	Memory impairment, concentration and attention impairment, executive dysfunction, anxiety, depression history of panic attack, history of suicidal ideation, persistent headaches.	Attention impairment, executive dysfunction, anxiety, depression	Executive dysfunction, history of panic attack	Cognitive impairment, executive dysfunction, mood disturbances, persistent headaches
			Diagnosed with ADHD, GAD, and PDD and currently on medication.			
Outcome measures	Cognitive function, executive function, social isolation	Executive function, anxiety, depression, irritability	Cognitive function, executive function, anxiety, depression, insomnia			

ADHD: Attention deficit hyperactivity disorder; AU: arbitrary unit; GAD: Generalised anxiety disorder; GMHAT: Global Mental Health Assessment; M: male, MCI: mild cognitive impairment (according to assessment cut-off); PROMIS: Patient-Reported Outcomes Measurement Information System Health Assessment Questionnaire—Physical Function 24a; PDD: Persistent depressive disorder; PTP: Participant; SLUMS: Saint Louis University Mental Status; TBI: traumatic brain injury; WNL: within normal limits (according to assessment cut-off).

**Fig 2:**
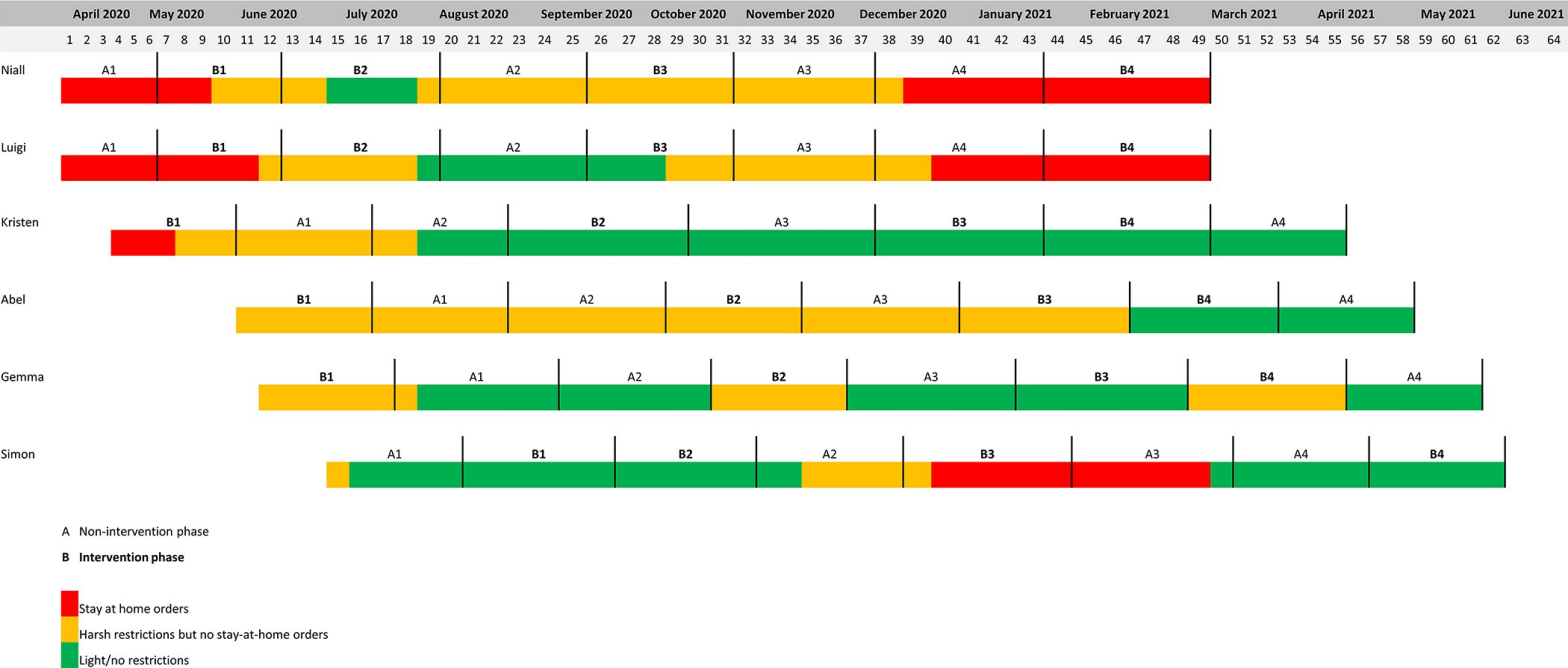


Fig 2 illustrates a complete study timeline across all participants along with the sequence of intervention (B) and non-intervention (A) phases. This figure also provides key information on the COVID pandemic and the restrictions in place for each participant at the time of data collection.

**Fig 3:**
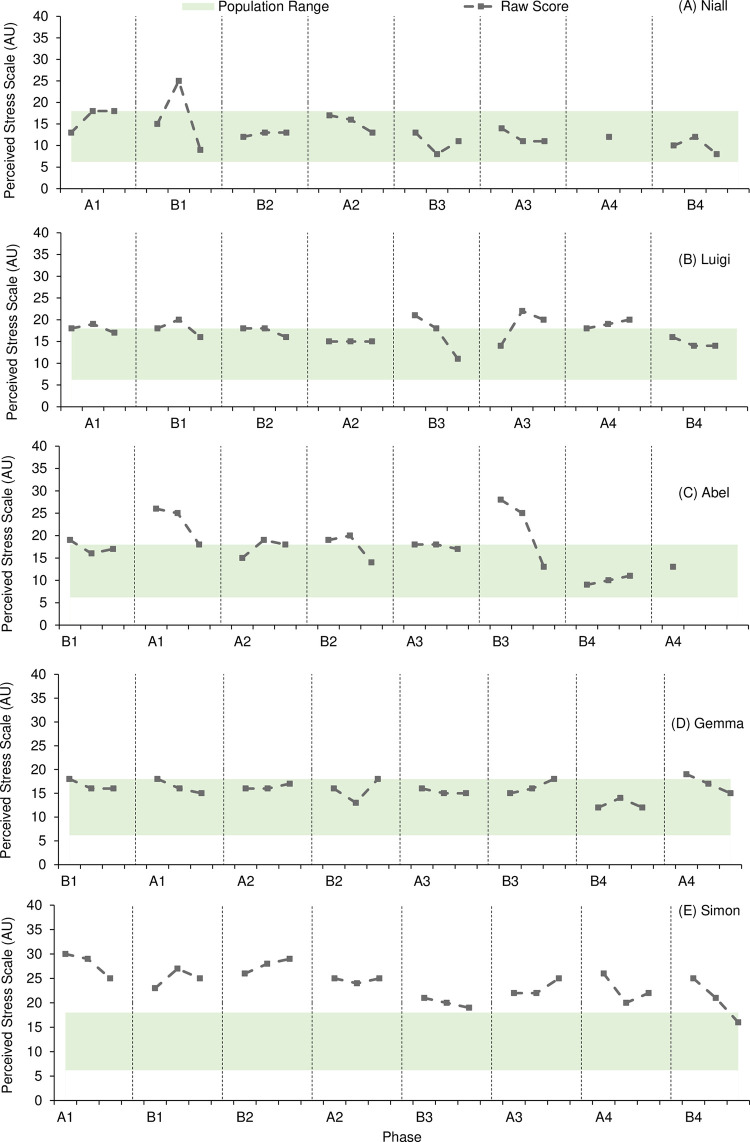


Fig 3 presents results from the PSS assessment.

### Participant activity levels

Participant were habitually active during the intervention engaging in various forms of physical activity including leisure, exercise, and sport. Table 2 presents a summary of the activities reported by participants during both intervention (B) and non-intervention (A) phases with bolded text being the prescribed activities.

**Table 2:** Summary of self-selected physical activities (normal) and intervention (bold).

	Phase A	Phase B
Niall	1. Calisthenics, inconsistent walks & runs 2. Low-intensity gym-based workout, hockey training* & 1. Hockey training* & matches 3. Activity log not available	**2. Resistance & cardiovascular activity**, calisthenics, walks*, hockey training **3. Resistance & cardiovascular activity**, five-a-side football, hockey training* 4. Resistance & cardiovascular activity, walks, runs, gym workouts, hockey training & matches **4. Resistance & cardiovascular activity**, calisthenics, walks*, hockey training
Luigi	1. Treadmill runs, playing with kids (outdoors), therapeutic exercises (injury) 2. Treadmill runs, playing with kids (outdoors), moderate outdoor manual labour 3. Treadmill runs, home workout 4. Treadmill runs, calisthenics	**1. Resistance & cardiovascular activity**, rollerblading, playing with kids (outdoors), calisthenics **2. Resistance & cardiovascular activity** **3. Resistance & cardiovascular activity** **4. Resistance & cardiovascular activity**
Kristen	1. Heavy housework, outdoor walks 2. Heavy housework 3. Activity log not available 4. Moderate housework	**1. Resistance & cardiovascular activity** **2. Resistance & cardiovascular activity**, outdoor walks, moderate-heavy housework **3. Resistance & cardiovascular activity**, moderate housework, therapeutic exercise (post-surgery) **4. Resistance & cardiovascular activity**, moderate housework, snowmobiling
Abel	1. Camp activities, moderate outdoor manual labour, outdoors walks 2. Recreational sport activities, outdoor walks 3. Camp activities, outdoor walks 4. Light outdoor manual labour, indoor walks	**1. Resistance & cardiovascular activity**, camp activities, moderate outdoor manual labour, outdoor walks **2. Resistance & cardiovascular activity**, recreational sport activities, outdoor walks **3. Resistance & cardiovascular activity**, recreational sport activities, outdoor walks **4. Resistance & cardiovascular activity**, Light outdoor manual labour, indoor walks
Gemma	1. Hiking, outdoor runs*, circuit training* 2. Outdoor walks, recreational outdoor activities, gym class* 3. None reported 4. Light intensity calisthenics	**1 Resistance & cardiovascular activity**, interval training, circuit training, rehabilitation exercises **2. Resistance & cardiovascular activity**, light-intensity calisthenics, yoga **3. Resistance & cardiovascular activity**, yoga **4. Resistance & cardiovascular activity**, light-intensity calisthenics
Simon	1. Cycling*, resistance training, runs 2. Resistance training, runs 3. Resistance training, runs 4. Resistance training, runs	**1. Resistance & cardiovascular activity**, touch rugby*, cycling, resistance training, runs **2. Resistance & cardiovascular activity**, cycling*, runs **3. Resistance & cardiovascular activity**, cycling*, resistance training, runs **4. Resistance & cardiovascular activity**, runs

Additional activities were informed by daily activity log or included follow-up interviews. The number represents the phase ordering (e.g., 1 = first A or B phase).

* Indicates an inconsistent involvement in the stated activity.

### Quantitative analysis

Table 3 provides a summary of the six individual cases, including visual and statistical analysis, across each symptom of interest. Individual case results can be found in S3–S8 Tables. The effect that active rehabilitation had on symptoms of motor function was not reported as no participants included in the study had any measurable motor impairments.

**Table 3:** Summary of quantitative case results.

Outcome measure	Visual analysis	WC-SMD	NAP	Summary		
Cognitive function	4.50–5.75 (small—moderate)	-1.14–0.30 (trivial—large)	0.25–0.59	1	1	1
Executive function	4.00–7.00 (small—large)	-0.18–1.69 (trivial—large)	0.44–0.79	4	2	0
Mindful attention	3.50–4.75 (small)	-0.52–0.09 (trivial—moderate)	0.30–0.51	0	1	1
*Total number–overall cognitive function*				5	6	1
Loneliness	6.50 (moderate)	-0.01 (trivial)	0.40	0	1	0
Anxiety	5.00–6.50 (moderate)	-0.87–0.26 (trivial—large)	0.25–0.53	1	1	2
Depression	3.50–4.75 (small)	-0.58–1.01 (mod.—large)	0.26–0.75	1	0	3
Irritability	7.00 (large)	0.11 (trivial)	0.52	0	1	0
Sleep	0 (none)	-0.20 (small)	0.43	0	1	0
*Total number–overall mood and behaviour*				2	4	5

A = non-intervention phase. B = intervention phase. NAP = non-overlap of all pairs. SD = standard deviation. WC-SMD–within case standardized mean difference. Green = positive effect. Yellow = unclear effect. Red = negative effect.

**Fig 4:**
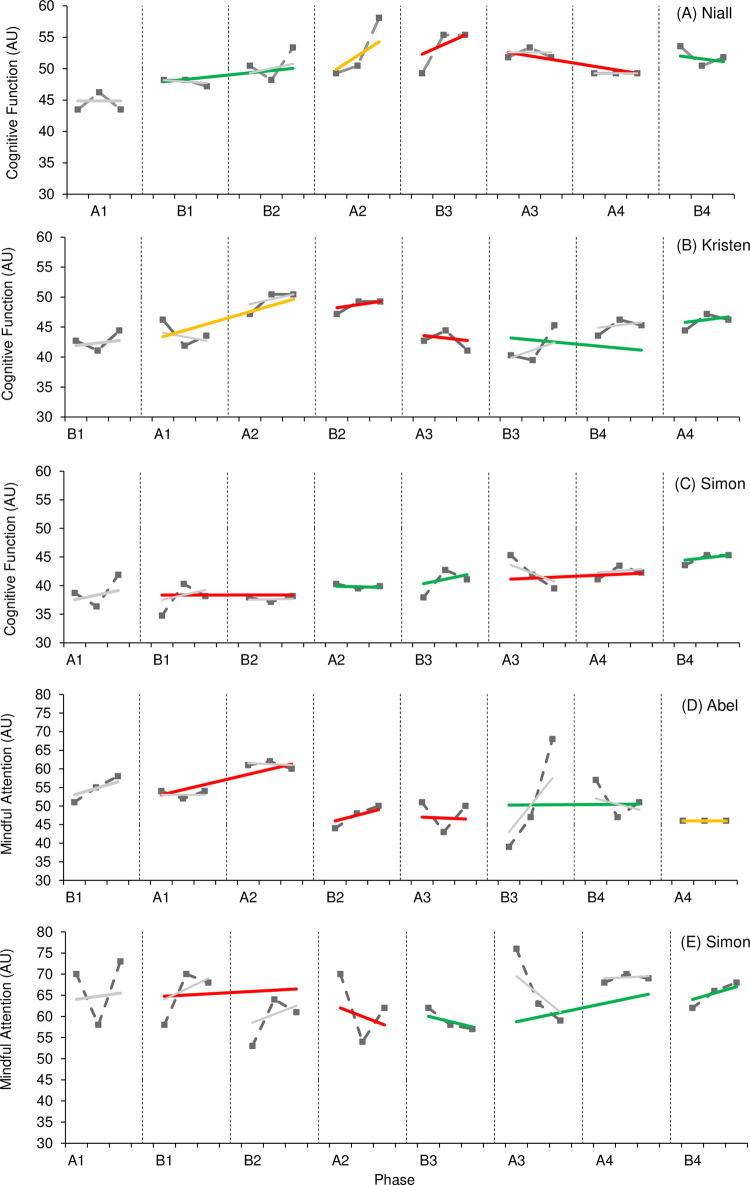


**Fig 5:**
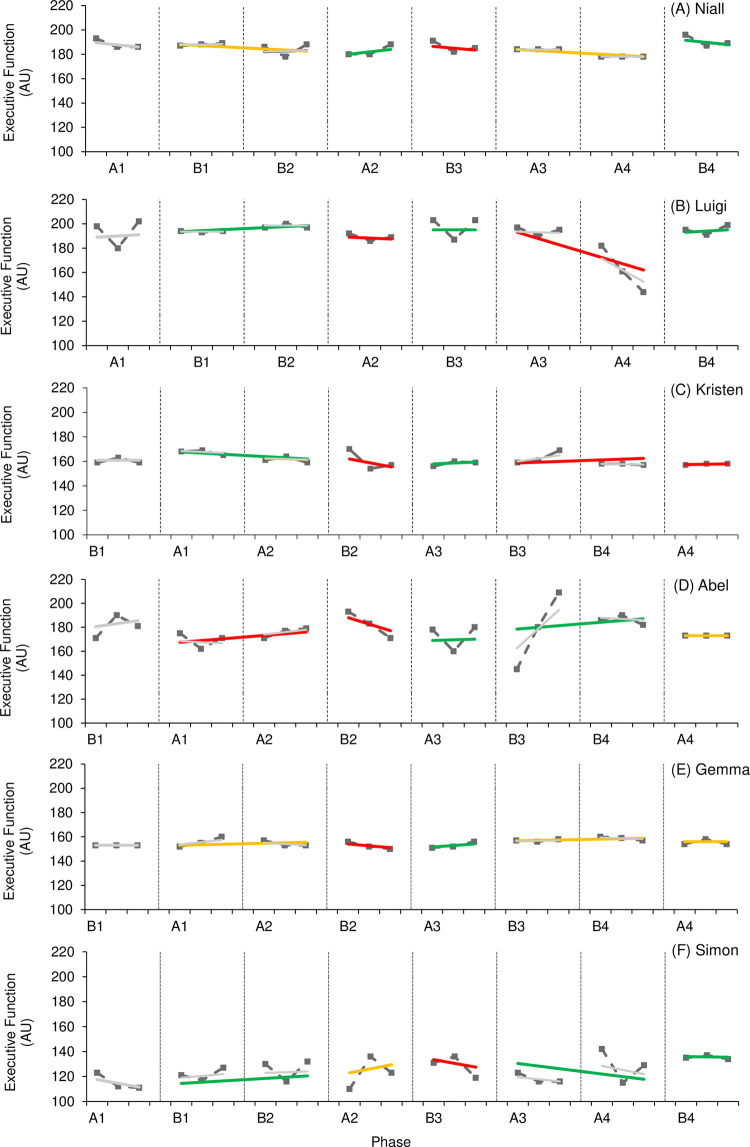


Figs 4 and 5 provide visual results of outcome measures related to cognitive function. Effects on general cognitive function (Fig 4) varied, with one participant (Niall) demonstrating a positive effect, one participant (Kristen) demonstrating a negative effect, and one participant (Simon) demonstrating an unclear effect. It should be noted that levels of general cognitive function consistently demonstrated an upward trend across intervention phases throughout the study for Simon, indicating a potential positive effect; however, statistical analysis supports an unclear effect. Visual and statistical analysis observed consistent overlap in all three cases as well. Levels of cognitive function were higher in the final phase for all three cases compared to those levels reported in the first study phase. Effects on mindful attention (Fig 4) also varied, with one case demonstrating negative visual and statistical analysis (Simon) and one demonstrating a small visual but trivial statistical effect (Abel). High levels of variability throughout the study should be noted here along with consistent overlap between phases; therefore, it is difficult to determine the effect active rehabilitation had on levels of mindful attention.

Though the WC-SMD ranged from trivial to large, four of the six participants demonstrated a positive effect (Niall, Luigi, Abel, Simon) for measures of executive function (Fig 5). Three of these participants (Niall, Luigi, Simon) also reported higher levels of executive function at the final phase of the study compared to the start of the study. Only Abel reported worse scores during the final phase of the study; however, a large WC-SMD (1.69) and NAP value of 0.79 suggests a positive effect of active rehabilitation overall. It should be noted that Kristen consistently demonstrated decreased variability of scores in non-intervention phases indicating more stable results for executive function in the absence of an active rehabilitation programme. This would suggest that the presence of an active rehabilitation programme had the potential to influence levels of outcome measures, albeit with a lack of statistical certainty. All cases demonstrated consistent overlap with visual analysis; however, NAP values in three cases (Niall, Abel, Simon) demonstrated a higher probability of increased executive function in intervention phases.

**Fig 6:**
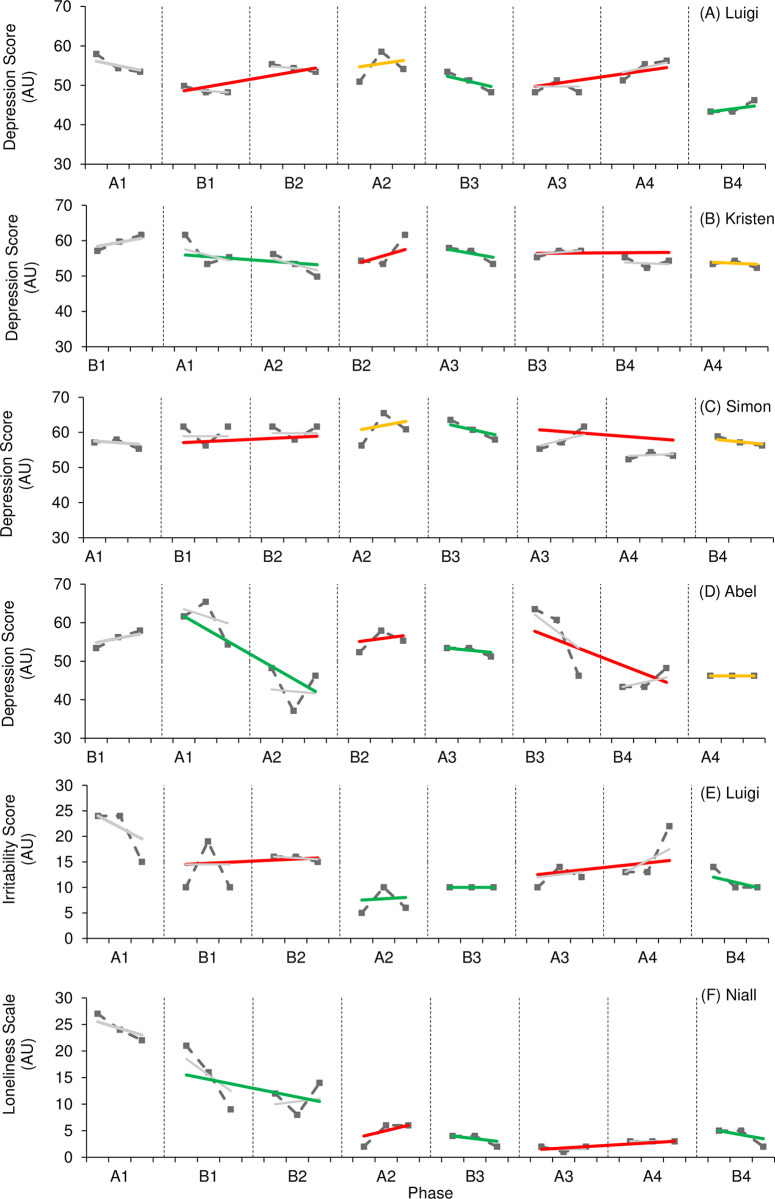


**Fig 7:**
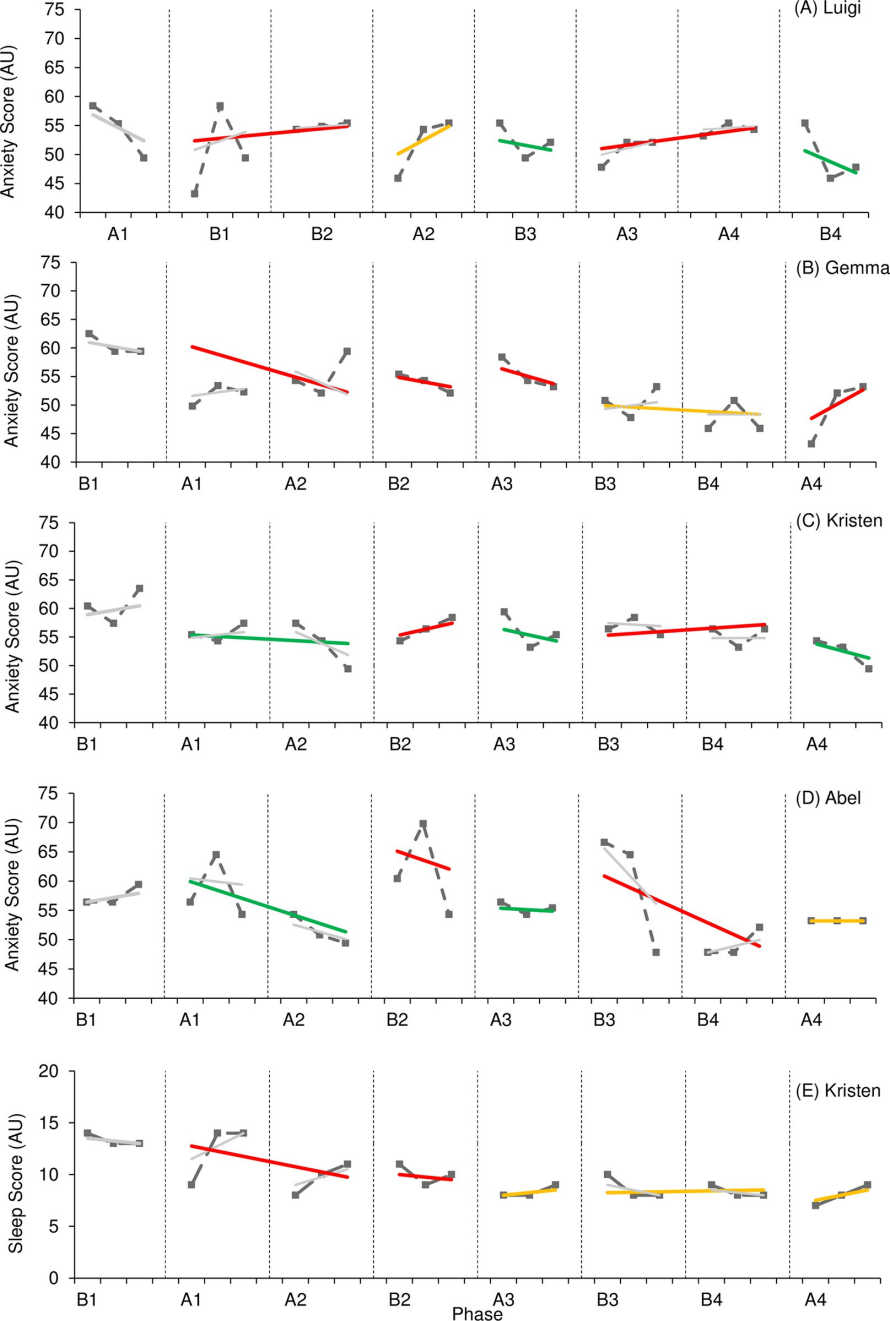


Figs 6 and 7 provide visual results of outcome measures related to mood and behaviour. The effect of active rehabilitation on outcome measures considered ‘core clinical features’ according to TES clinical criteria (depression, irritability, social isolation/loneliness) (Fig 6) varied. One participant (Luigi) demonstrated a large positive effect on levels of depression, supported by both visual and statistical analysis; however, three participants (Kristen, Abel, Simon) demonstrated a small, negative effect on levels of depression. Interestingly, all participants aside from one (Simon) reported lower levels of depression during the final phase of the study compared to initial levels. There was no consistency of patterns in variability or trend, but overlap between phases was consistently present. NAP values also varied, indicating a variation in the likelihood of a randomly selected point taken during an intervention phase resulting in lower depression scores. Only one participant reported symptoms of loneliness and social isolation (Niall), and one participant reported symptoms of irritability (Luigi). In both instances, visual analysis indicated a positive effect. Levels of loneliness demonstrated a consistent downward trend during intervention phases despite increased variability. It should also be noted that Luigi demonstrated decreased variability for levels of irritability across intervention phases. A positive effect is further supported by improved levels reported during the final phase of the study compared to initial levels, as well as a general decline observed across the entire study; however, statistical analysis reports a trivial effect in both outcome measures.

The effect of active rehabilitation on outcome measures considered ‘supportive clinical features’ according to TES clinical criteria (anxiety, sleep quality/insomnia) (Fig 7) also varied. One participant (Luigi) reported a small-to-moderate effect on levels of anxiety, supported by visual and statistical analysis; however, Kristen and Abel reported a moderate, negative effect. The effect of active rehabilitation on Gemma’s level of anxiety was unclear. Visual analysis demonstrated a small effect; however, statistical analysis reports the effect as trivial. Interestingly, all participants demonstrated reduced levels of anxiety during the final phase of the study compared to those levels reported during the initial phase; however, overlap was present between all phases across all cases suggesting a temporal pattern of change. Only Kristen reported NAP levels which could suggest a greater probability of lower levels of anxiety in non-intervention phases (NAP = 0.25). It should be noted that Gemma consistently demonstrated a downward trend in levels of anxiety in intervention phases. Only one participant reported symptoms of reduced sleep quality and insomnia (Kristen). Despite WC-SMD reporting a small negative effect, visual analysis reported a trivial effect. Further, NAP suggests the probability randomly selected data point for sleep score being lower during an intervention phase was 43%. It should be noted that Kristen’s sleep score was lower at the end of the study compared to those reported at the start of the study, indicating an improvement in symptoms.

### Qualitative data

**Fig 8:**
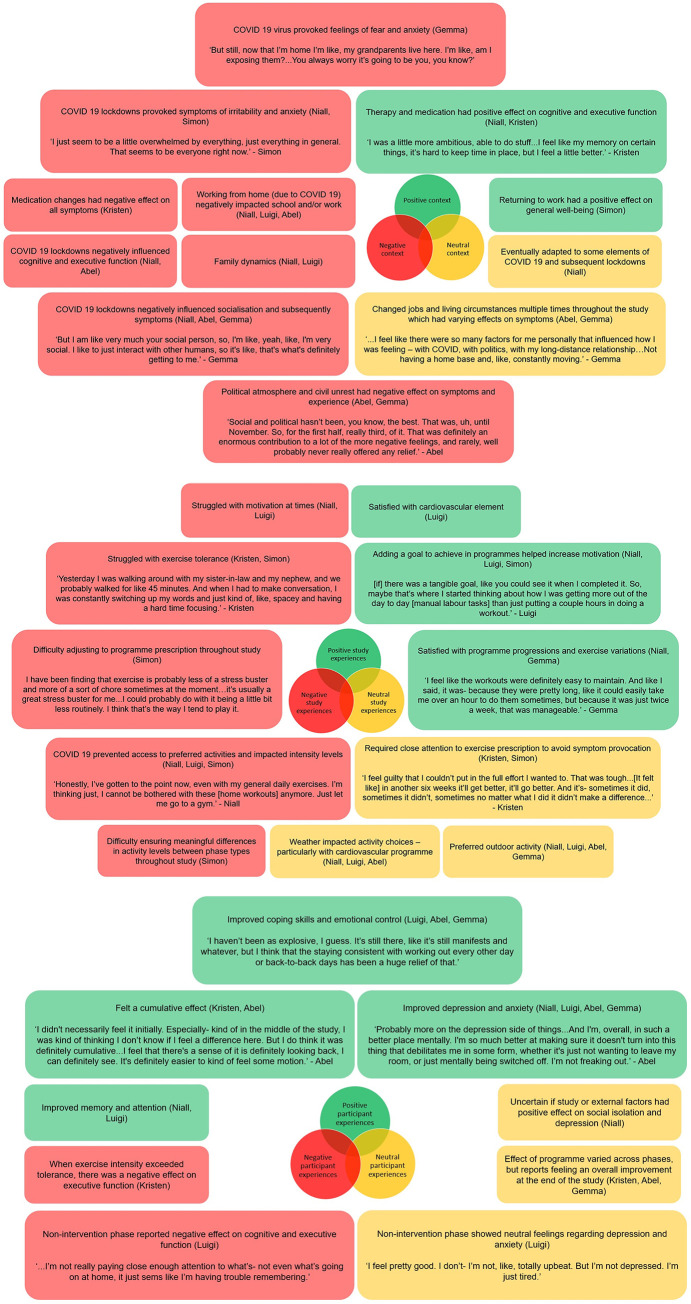


Fig 8 presents a visual summary of the topics emerged from the semi-structured interviews, including information relevant to the context of the study (8A), intervention experience (8B), and perception towards the effect of the intervention on symptoms (8C). Topics of discussion highlighted in green present information that may have contributed to a positive participant experience and generally included improved memory, coping skills emotional control and anxiety, the role of goal setting, and satisfaction with the active rehabilitation programme. Topics of discussion highlighted in red present a potentially negative experience. These include information such as COVID-19 and the political atmosphere as well as exercise tolerance, progression, and motivation. Topics of discussion highlighted in yellow presented points that were not clearly linked to a positive or negative experience (e.g., weather, unclear or neutral feelings, and preferences).

The most obvious influence that emerged from the results which impacted every participant was the presence of the COVID-19 pandemic and subsequent lockdown restrictions. This seemed to influence Niall and Luigi the most. These two participants began the study at the very start of the pandemic where restrictions were the harshest and the levels of uncertainty were the highest. It seemed to affect Kristen the least, who lives in a quite secluded part of New York (state). The presence of the pandemic had both a direct and indirect effect on the success of the intervention. The pandemic influenced some outcomes of interest such as symptoms of anxiety and depression. Lockdown restrictions could also influence executive function as illustrated by Niall, who reported that motivation, attention, and concentration were disrupted. It should be noted that two participants contracted COVID-19 during the study (Kristen, Abel), resulting in further direct effects of the global pandemic. Indirectly, COVID-19 and subsequent lockdowns also impacted socialisation which had a notable impact on Niall, Abel, and Gemma. That said, we do also acknowledge that the pandemic and ‘stay at home’ messaging did facilitate a home-based active rehabilitation programme whereby greater leisure time was available for some due to reduced travel to and from working and social commitments.

The political discourse present in the United States affected the three American participants to varying degrees. Abel considers himself passionate about, and therefore hyper-involved in, the political culture of the US. Therefore, the BLM protests, the Capitol attack, and the Presidential election resulted in increased feelings of stress. This effect was vastly reduced in the second half of the study following the end of the election cycle, as evidenced in the PSS scores. While still affected by the same factors, Gemma was less involved and therefore found that the political discourse had more of an ‘exhausting’ effect and produced feelings of irritability. Finally, Kristen reported some levels of stress concerning the present circumstances; however, she was better able to cope as she was not as directly exposed to some of the unrest living in a secluded area compared to Abel and Gemma who were more city-based.

Work was another factor that was mentioned by several participants. As someone working in the healthcare industry, who also received a work promotion during the study, Luigi felt direct pressure from the COVID-19 pandemic. This was evidenced in his PSS scores. Abel and Gemma both suffered from job instability. Abel changed jobs at least three times during the study, as each position was more seasonal in nature and induced a degree of anxiety and frustration as well as resulting in periods of physically demanding work. Gemma’s work was directly impacted by the COVID-19 pandemic. After the onset of the pandemic, her job position became less stable. She tried taking on per diem work, but regardless her work situation was unstable largely throughout the study. In contrast, Simon reported a positive effect on his general well-being after returning to work following a prolonged period of medical leave.

Programme prescription and activity selection was another factor that emerged from the data. Exercise intolerance was present in two participants (Kristen, Simon) who both reported a history of moderate-to-severe TBI. Kristen struggled with fully participating in the study as a direct result of her exercise intolerance. While she was willing to ‘push through’ some short-term adverse reactions, she expressed that any time she overcame her exercise intolerance, the phase would change to a non-intervention phase and she had to start all over again during the next intervention phase. This sentiment is further supported by reported outcome measures in phase B4, which occurred immediately after phase B3 resulting in a double intervention phase. When given an additional six weeks of intervention, levels were improved for cognitive function, anxiety, and depression. Simon’s exercise intolerance was less severe, but it did have an impact on what activities could be prescribed. He did not report any adverse events because of the programme, aside from his difficulty with constant disruption and lack of control over his programmes. COVID-19 and subsequent lockdowns resulted in the disruption of preferred activities for several participants. Niall was not able to go to the gym or consistently take part in field hockey which he reported was a source of frustration. Not only were these his preferred activities, but both involved an element of socialisation and associated social capital. Luigi noted that he struggled to find ‘me time’ as he never felt comfortable returning to the gym even when it was open despite this being his preferred setting due to a fear of contracting COVID-19. Simon struggled with the inconsistency of gym availability which resulted in constant changes to his programmes. Finally, the weather had both a positive and a negative impact on activity selection depending on the time of year. Niall, Luigi, Abel, and Gemma all preferred outdoor activities, which meant participants enjoyed activity more in the warmer and less rainy months of the year when they could get outside. Despite these factors, various participants still expressed satisfaction with their programmes (Niall, Luigi, Gemma). Positive experiences were increased when the intervention utilised a goal-setting element to increase motivation (Niall, Luigi, Simon).

## Discussion

This mixed-methods single case design provides the first primary level evidence to suggest that active rehabilitation has potential as an intervention for those suffering from symptoms related to suspected CTE. Overall, active rehabilitation had a largely positive effect on measures of cognitive function whilst the results for mood and behaviour across the intervention period was mixed and heavily influenced by factors such as the COVID-19 pandemic.

Quantitative analysis suggests that active rehabilitation had an almost equally positive and unclear effect on overall cognitive function. These findings reflect that reported by Hearn et al. [9] who observed a pooled estimate across three studies as moderately positive but with confidence intervals crossing positive, unclear, and negative effects on cognitive function in populations with tau pathologies. Considering the outcome measures of general cognitive function (one positive, one trivial, one negative), the effect of active rehabilitation was inconclusive and suggestive of individual variation. The effect of active rehabilitation on measures of general cognitive function were inconclusive, with one trivial effect and one negative effect reported. Across the study, the only impairment observed in all participants was executive dysfunction. This supports the inclusion of memory impairment and executive dysfunction in the updated TES criterion presented by Katz and colleagues [5]. Four of the six participants reported a positive effect for executive function overall (Niall, Luigi, Abel, Simon) with two others being unclear (Kristen, Gemma). Importantly, there was no negative effect observed on measures of executive function. Overall, the effect of active rehabilitation on cognitive function has yet to be fully determined but is promising. While the underlying physiological mechanisms are yet to be fully understood, exercise is known to have a positive impact on neurogenesis and angiogenesis [19, 20] which can have a positive influence on executive function.

The results for mood and behaviour symptoms were largely inconclusive. When considering the individual outcome measures, one positive, one trivial, and two negative effect were observed for anxiety. Whilst this outcome is surprising from a purely quantitative viewpoint, based on the qualitative results, it is unlikely that these effects are attributed solely to the active rehabilitation programme. For example, the qualitative data suggests COVID-19 and subsequent lockdowns had both a direct and indirect effect on many of these outcomes. Participants expressed how the consequences of lockdowns in response to the pandemic directly influenced symptom levels. Specifically, lockdowns negatively influenced components of executive function, as well as levels of anxiety and depression. One participant (Gemma) further expressed her feelings of fear and anxiety about the presence of the virus itself, specifically about the danger it posed to her loved ones. These findings reflect similar observations in various populations where the COVID-19 pandemic resulted in higher reported levels of stress, anxiety, and depression [21, 22]. Supported by the PSS scores and participant interviews, the influence was likely stronger earlier on in the intervention period as participants eventually began to adapt to life with COVID-19. For those who contracted the virus (Kristen, Abel), symptom levels were also likely influenced by the presence of the virus. In addition to the respiratory and inflammatory symptoms associated with COVID-19, contracting COVID-19 has also been associated with the development of fatigue, anxiety, depression, and cognitive disturbances [23].

Despite the various proximal and distal factors being discussed, some of which were unprecedented and, at times, had a substantial influence on participant symptoms, this study was still able to provide positive results. Supported by the quantitative results, participants directly reported that they felt the active rehabilitation programme had a positive effect on symptoms of memory, attention, depression, and anxiety. It was suggested the effect may have been cumulative across the twelve-month intervention period (Kristen, Abel, Gemma). This is supported by the improvement of various outcome measures at the end of the study despite conflicting visual and/or statistical analysis, such as the improvement in levels of cognitive function in the case of Kristen or the improvement in levels of depression in the case of Abel. At the very least the programme offered a way to cope with or better manage symptoms as suggested by Luigi, Abel, Gemma. This is demonstrated by the consistent decreased variability in levels of irritability (Luigi) and loneliness (Niall) consistently observed during intervention phases despite a reported trivial effect. If repeated under more stable conditions, there is a potential for a greater, and perhaps stronger, number of observed positive effects, especially when considering the greater understanding of the context and individual preferences that emerged in this study. Further, some of the participants may not have met the eligibility criteria had these unprecedented factors not been present. For example, Niall’s levels of loneliness were only above reported population average during the baseline phase and proceeded to drop throughout the first half of the study independent of the active rehabilitation programme. Therefore, future research and clinical practice might consider symptoms and inclusion criteria that are evident outside of other factors such as the environment and personal circumstances.

This study also supports the use of a person-centred approach in future research and clinical care, as evidenced by the enhanced understanding of participant context and study experience. Utilising a mixed methods approach with data integration has allowed for an in-depth understanding of the observed visual and statistical results. This was further enhanced by the study length (twelve months). A person-centred approach to programme prescription ensured participant satisfaction and reduced dropouts. This is evidenced by the six participants who completed the twelve-month study and reported programme satisfaction despite many not having access to preferred activities.

Whilst this study is the first empirical investigation within this area, this study is not without some limitations. Firstly, we note that some features of the study might limit the generalisability of the findings. Indeed, the TES criteria was updated after the study commenced, the study was carried out during a pandemic, and the outcomes lack specificity to a a suspected CTE population. Secondly, whilst online methods were essential for the success of this study, there is an inherent lack of control over aspects such as other activities and the intensity these are performed which would impact some outcomes used in the study. Also, the online nature meant that outcomes were limited to questionnaires and qualitative data whereas objective measure to supplement these might have strengthened the validity of these findings. Future research should continue to utilise a person-centred approach within this area of enquiry to improved intervention efficacy while continuing to maintain study adherence and participant satisfaction. Also, future work should seek substantiate the findings in this study to allow for pooling of data.

In conclusion, this study has provided evidence to establish the potential use of active rehabilitation for the management of suspected CTE using a mixed-methods single case research design. Based on the results of this study, a narrative summary of the integrated qualitative and quantitative results has been provided in Table 4. This table provides a holistic understanding of individual cases and observed effects supporting an overall conclusion, though we do refer readers to the individual cases in S3–S8 Tables given the person-centred approach of the study. This study has offered preliminary evidence which suggests active rehabilitation may offer some benefit to individuals with symptoms of suspected CTE and warrants further investigation using standardised and innovative methodologies. This study has also demonstrated the benefit of a person-centred approach to both clinical research and practice. Considering factors such as personal circumstances, cultural climate, and a detailed intervention response allows for a better understanding of an intervention effect within the context of the study.

**Table 4:** Summary of integrated results.

Participant	Summary of observed effect–Cognitive Functioning
Niall	Positive. Particularly on memory and attention. Effect was boosted by coping mechanisms developed with the help of NHS CBT. Proximal and distal factors had a negative effect on both outcome measures and programme delivery, especially COVID-19 lockdowns.
Luigi	Positive. Particularly on memory. Proximal and distal factors, especially COVID-19 lockdowns, did not influence the effect of the programme but perhaps disrupted the size of effect reported.
Kristen	Inconclusive. Executive function particularly sensitive to medication levels. Exercise intolerance had a significant impact on outcome measures.
Abel	Positive. Quantitative results for attention were inconclusive; however, absence of intervention showed consistent negative effect. Expressed feeling a cumulative effect from the intervention.
Gemma	Inconclusive. Qualitative analysis offered little insight to support any effect.
Simon	Positive. Qualitative analysis offered little insight.
Participant	Summary of observed effect–Mood & behavioural symptoms
Niall	Inconclusive. Proximal and distal factors had a negative effect on both outcome measures and programme delivery, especially COVID-19 lockdowns. Unsure if intervention was fully responsible for perceived improvement of loneliness and social isolation. Adding a social element to intervention had a positive effect on outcome measures.
Luigi	Positive. Learned to use activity as a coping mechanism for negative mood/behavioural symptoms, particularly irritability. The absence of intervention contributed to increased feelings of apathy and lack of control, whereas presence of intervention reported increased control and positive emotions alongside improved outcome measures. Strong association between PSS scores and mood/behavioural outcome measures. Proximal and distal factors, especially COVID-19 lockdowns, did not influence the effect of the programme but perhaps disrupted the size of effect reported.
Kristen	Negative effect on anxiety and depression. Depression and apathy particularly sensitive to medication levels. Exercise intolerance had a significant impact on outcome measures. Feelings of isolation caused by COVID-19 pandemic and civil unrest also contributed. No effect observed on sleep.
Abel	Inconclusive. Despite negative quantitative analysis, expressed feeling a cumulative effect and general improvement in symptoms. Particularly on levels of depression. Proximal and distal factors had a significant influence on mood/behavioural symptoms, including work, travel, COVID-19 pandemic, and civil unrest. Negative effects primarily observed in first half of study.
Gemma	Inconclusive. Expressed feeling a cumulative effect particularly with levels of anxiety. COVID-19, civil unrest, and frequent travel had a particular influence on levels of anxiety, stress, and feelings of isolation.
Simon	Inconclusive. Quantitative analysis offered a negative effect; however, qualitative analysis offered little insight.

## Supporting information

S1 Table Screening assessment. MCI: mild cognitive impairment; PROMIS: Patient-reported outcomes measurement information system. N/A: not applicable due to multiple subscales used.
(DOCX)

S2 Table Outcome assessments. (DOCX)

S3 Table Niall’s summary of results. (DOCX)

S4 Table Luigi’s summary of results. (DOCX)

S5 Table Kristen’s summary of results. (DOCX)

S6 Table Abel’s summary of results. (DOCX)

S7 Table Gemma’s summary of results. (DOCX)

S8 Table Simon’s summary of results. (DOCX)
