# Supplementary material for: The effects of person-centred active rehabilitation on symptoms of suspected Chronic Traumatic Encephalopathy: A mixed-methods single case design
Source: PLoS One. 2024 May 30;19(5):e0302260. doi: 10.1371/journal.pone.0302260 (PMC11139304; doi:10.1371/journal.pone.0302260)
Supplement: S1 Table — MCI: mild cognitive impairment; PROMIS: Patient-reported outcomes measurement information system. N/A: not applicable due to multiple subscales used. (DOCX) [file pone.0302260.s001.docx]

| **S1.** Screening Assessments | |
| --- | --- |
| Assessment | Description |
| Saint Louis University Mental Status (SLUMS) | - 11-item cognitive screening assessment - Total possible score: 30 AU - Optimal MCI cut-off: 25 AU - Higher scores indicate higher levels of cognitive function |
| Global Mental Health Assessment Tool (GMHAT) | - Global mental health screening assessment - Total possible score: Not applicable as the full tool was not used. - Impairment cut-off: Not available - Higher scores indicate higher levels of mood/behaviour impairment (e.g., higher levels of anxiety, higher levels of depression) |
| PROMIS bank v2.0-Physical Function 24a | - 24-item physical function screening assessment - Total possible score: 102 AU - Cut-off for impairment: 98 AU - Higher scores indicate higher levels of motor function |
