# Supplementary material for: The effects of person-centred active rehabilitation on symptoms of suspected Chronic Traumatic Encephalopathy: A mixed-methods single case design
Source: PLoS One. 2024 May 30;19(5):e0302260. doi: 10.1371/journal.pone.0302260 (PMC11139304; doi:10.1371/journal.pone.0302260)
Supplement: S2 Table — (DOCX) [file pone.0302260.s002.docx]

| **S2.** Outcome assessments | | |
| --- | --- | --- |
| **Area of assessment** | Scale | Description |
| **Cognitive function** | PROMIS Short Form v2.0 - Cognitive function 8a | - 8-item cognitive function self-report assessment - Highest possible t-score: 63.5 AU - Cut-off for impairment: 45 AU - Assessed components of global cognitive function - Higher scores indicate higher levels of cognitive function |
|  | Executive Skills Questionnaire (ESQ) | - 12-item executive function assessment - Total possible score: 252 AU - Cut-off for impairment: N/A - Assessed 12 components of executive function such as sustained attention, working memory, and metacognition - Higher scores indicate higher levels of executive function |
|  | Mindful Attention Awareness Scale (MAAS) | - 15-item mindful attention assessment - Total possible score: 90 AU - Population average: 58 ± 10 AU - Assessed objective experiential awareness and attention - Higher scores indicate higher levels of attention |
| **Mood/ behavioural changes** | PROMIS Short Form v1.0 - Anxiety 8a | - 8-item anxiety self-report assessment - Highest possible t-score: 83 AU - Cut-off for impairment: 55 AU - Assessed components of anxiety such as worry, fear, and feeling on edge - Higher scores indicate higher levels of anxiety |
|  | PROMIS Short Form v1.0 - Depression 8b | - 8-item depression self-report assessment - Highest possible t-score: 81 AU - Cut-off for impairment: 55 AU - Assessed components of depression such as hopelessness and distress - Higher scores indicate higher levels of depression |
|  | Brief Irritability Test (BITe) | - 5-item irritability assessment - Total possible score: 25 AU - Population average: 13 ± 5.5 AU - Assessed components of irritability such as anger, hostility, and neuroticism - Higher scores indicate higher levels of irritability |
|  | UCLA Loneliness Scale | - 20-item loneliness assessment - Total possible score: 60 AU - Population average: 33 ± 7.5 AU - Assessed components of loneliness such as social isolation, depression, and interpersonal relationships - Higher scores indicate higher levels of loneliness |
|  | Pittsburgh Sleep Quality Index (PSQI) | - 7-item sleep quality assessment - Total possible score: 21 AU - Cut-off for impairment: < 5 AU - Assessed components of sleep quality such as latency, efficiency and disturbances - Higher scores indicate worse sleep quality |
