# Supplementary material for: The effects of person-centred active rehabilitation on symptoms of suspected Chronic Traumatic Encephalopathy: A mixed-methods single case design
Source: PLoS One. 2024 May 30;19(5):e0302260. doi: 10.1371/journal.pone.0302260 (PMC11139304; doi:10.1371/journal.pone.0302260)
Supplement: S3 Table — (DOCX) [file pone.0302260.s003.docx]

| **S3**. Niall’s summary of results | | | | | | | |
| --- | --- | --- | --- | --- | --- | --- | --- |
| Outcome measure | Visual analysis | Mean A ± SD | Mean B ± SD | Mean ∆ | WC-SMD (95%CI) | NAP (95%CI) | Effect summary |
| Cognitive function | 4.75 (small) | 49.6 ± 4.1 | 50.9 ± 2.9 | 1.3 | 0.30 (small)  (-1.35, 1.83) | 0.59  (0.36, 0.78) |  |
| Executive function | 4.00 (small) | 183.3± 4.7 | 187.2± 4.5 | 3.92 | 0.79 (moderate)  (-0.01, 1.57) | 0.76  (0.53, 0.90) |  |
| Loneliness | 6.50 (moderate) | 8.42 ± 9.8 | 8.50 ± 6.1 | 0.08 | -0.01 (trivial)  (-0.63, 0.61) | 0.40  (0.21, 0.62) |  |

Desired effect. Undesired effect. Trivial effect/Overlap. A = non-intervention phase. B = intervention phase. NAP = non-overlap of all pairs. SD = standard deviation. WC-SMD – within case standardized mean difference. 95%CI = 95% confidence interval. ∆ = mean difference.
