# Supplementary material for: The effects of person-centred active rehabilitation on symptoms of suspected Chronic Traumatic Encephalopathy: A mixed-methods single case design
Source: PLoS One. 2024 May 30;19(5):e0302260. doi: 10.1371/journal.pone.0302260 (PMC11139304; doi:10.1371/journal.pone.0302260)
Supplement: S4 Table — (DOCX) [file pone.0302260.s004.docx]

| **S4**. Luigi’s summary of results | | | | | | | |
| --- | --- | --- | --- | --- | --- | --- | --- |
| Outcome measure | Visual analysis | Mean A ± SD | Mean B ± SD | Mean ∆ | WC-SMD (95%CI) | NAP (95%CI) | Effect summary |
| Executive function | 7.00 (large) | 184.67 ± 16.47 | 196.08 ± 4.76 | 11.41 | 0.63 (moderate)  (0.03, 1.23) | 0.63  (0.03, 1.23) |  |
| Anxiety | 5.00 (moderate) | 52.8 ± 3.58 | 51.8 ± 4.62 | 1.00 | 0.26 (small)  (-0.60, 1.13) | 0.53  (0.32, 0.73) |  |
| Depression | 4.75 (small) | 53.28 ± 3.42 | 49.57 ± 4.08 | 3.71 | 1.01 (large)  (0.10, 1.92) | 0.75  (0.51, 0.89) |  |
| Irritability | 7.00 (large) | 14.00 ± 3.58 | 12.5 ± 3.29 | 1.50 | 0.11 (trivial)  (-0.50, 0.71) | 0.52  (0.31, 0.73) |  |

Desired effect. Undesired effect. Trivial effect/Overlap. A = non-intervention phase. B = intervention phase. NAP = non-overlap of all pairs. SD = standard deviation. WC-SMD – within case standardized mean difference. 95%CI = 95% confidence interval. ∆ = mean difference.
