# Supplementary material for: The effects of person-centred active rehabilitation on symptoms of suspected Chronic Traumatic Encephalopathy: A mixed-methods single case design
Source: PLoS One. 2024 May 30;19(5):e0302260. doi: 10.1371/journal.pone.0302260 (PMC11139304; doi:10.1371/journal.pone.0302260)
Supplement: S5 Table — (DOCX) [file pone.0302260.s005.docx]

| **S5**. Kristen’s summary of results | | | | | | | |
| --- | --- | --- | --- | --- | --- | --- | --- |
| Outcome measure | Visual analysis | Mean A ± SD | Mean B ± SD | Mean ∆ | WC-SMD (95%CI) | NAP (95%CI) | Effect summary |
| Cognitive function | 4.5 (small) | 45.49 ± 3.04 | 44.51 ± 3.23 | -0.98 | -1.14 (large)  (-2.30, 0.03) | 0.25  (0.11, 0.50) |  |
| Executive function | 5.5 (moderate) | 160.33 ± 4.81 | 161.17 ± 4.32 | -0.84 | -0.18 (trivial)  (-0.97, 0.61) | 0.44  (0.24, 0.66) |  |
| Anxiety | 6.50 (moderate) | 54.43 ± 2.98 | 57.22 ± 2.75 | -2.79 | -0.87 (large)  (-1.66, -0.08) | 0.25  (0.11, 0.48) |  |
| Depression | 4.75 (small) | 54.48 ± 3.06 | 56.59 ± 3.06 | -1.75 | -0.53 (moderate)  (-1.31, 0.24) | 0.33  (0.17, 0.57) |  |
| Sleep | None | 9.58 ± 2.31 | 10.08 ± 2.19 | -0.50 | -0.20 (small)  (-0.93, 0.53) | 0.43  (0.23, 0.65) |  |

Desired effect. Undesired effect. Trivial effect/Overlap. A = non-intervention phase. B = intervention phase. NAP = non-overlap of all pairs. SD = standard deviation. WC-SMD – within case standardized mean difference. 95%CI = 95% confidence interval. ∆ = mean difference.
