# Supplementary material for: The effects of person-centred active rehabilitation on symptoms of suspected Chronic Traumatic Encephalopathy: A mixed-methods single case design
Source: PLoS One. 2024 May 30;19(5):e0302260. doi: 10.1371/journal.pone.0302260 (PMC11139304; doi:10.1371/journal.pone.0302260)
Supplement: S6 Table — (DOCX) [file pone.0302260.s006.docx]

| **S6**. Abel’s summary of results | | | | | | | |
| --- | --- | --- | --- | --- | --- | --- | --- |
| Outcome measure | Visual analysis | Mean A ± SD | Mean B ± SD | Mean ∆ | WC-SMD (95%CI) | NAP (95%CI) | Effect summary |
| Executive function | 4.75 (small) | 172.67 ± 6.23 | 181.75 ± 15.4 | 9.15 | 1.69 (large)  (0.01, 3.38) | 0.79  (0.67, 0.91) |  |
| Mindful attention | 4.75 (small) | 51.25 ± 7.53 | 53.30 ± 6.31 | 2.05 | 0.09 (trivial)  (-0.82, 0.99) | 0.51  (0.30, 0.72) |  |
| Anxiety | 5.00 (moderate) | 55.90 ± 4.05 | 56.94 ± 7.48 | -2.04 | -0.58 (moderate)  (-1.78, 0.62) | 0.40  (0.22, 0.63) |  |
| Depression | 4.25 (small) | 50.78 ± 7.60 | 53.18 ± 6.68 | -2.4 | -0.29 (small)  (-1.00, 0.42) | 0.40  (0.21, 0.63) |  |

Desired effect. Undesired effect. Trivial effect/Overlap. A = non-intervention phase. B = intervention phase. NAP = non-overlap of all pairs. SD = standard deviation. WC-SMD – within case standardized mean difference. 95%CI = 95% confidence interval. ∆ = mean difference.
