# Supplementary material for: The effects of person-centred active rehabilitation on symptoms of suspected Chronic Traumatic Encephalopathy: A mixed-methods single case design
Source: PLoS One. 2024 May 30;19(5):e0302260. doi: 10.1371/journal.pone.0302260 (PMC11139304; doi:10.1371/journal.pone.0302260)
Supplement: S7 Table — (DOCX) [file pone.0302260.s007.docx]

| **S7 Table**. Gemma’s summary of results | | | | | | | |
| --- | --- | --- | --- | --- | --- | --- | --- |
| Outcome measure | Visual analysis | Mean A ± SD | Mean B ± SD | Mean ∆ | WC-SMD (95%CI) | NAP (95%CI) | Effect summary |
| Executive function | None | 154.58 ± 2.71 | 155.33 ± 3.08 | 0.75 | 0.26 (small)  (-0.55, 1.06) | 0.58  (0.35, 0.77) |  |
| Anxiety | 5.25 (small) | 52.98 ± 4.07 | 53.13 ± 5.38 | -0.13 | -0.03 (trivial)  (-0.84, 0.91) | 0.51  (0.30, 0.72) |  |

Desired effect. Undesired effect. Trivial effect/Overlap. A = non-intervention phase. B = intervention phase. NAP = non-overlap of all pairs. SD = standard deviation. WC-SMD – within case standardized mean difference. 95%CI = 95% confidence interval. ∆ = mean difference.
