# Supplementary material for: The effects of person-centred active rehabilitation on symptoms of suspected Chronic Traumatic Encephalopathy: A mixed-methods single case design
Source: PLoS One. 2024 May 30;19(5):e0302260. doi: 10.1371/journal.pone.0302260 (PMC11139304; doi:10.1371/journal.pone.0302260)
Supplement: S8 Table — (DOCX) [file pone.0302260.s008.docx]

| **S8 Table**. Simon’s summary of results | | | | | | | |
| --- | --- | --- | --- | --- | --- | --- | --- |
| Outcome measure | Visual analysis | Mean A ± SD | Mean B ± SD | Mean ∆ | WC-SMD (95%CI) | NAP (95%CI) | Effect summary |
| Cognitive function | 5.75 (moderate) | 40.85 ± 2.35 | 40.2 ± 3.41 | -0.65 | -0.10 (trivial)  (-0.36, 0.95) | 0.46  (0.25, 0.69) |  |
| Executive function | 4.5 (small) | 121.33 ± 7.72 | 127.92 ± 7.72 | 6.59 | 0.45 (small)  (-0.12, 1.01) | 0.75  (0.51, 0.89) |  |
| Mindful attention | 6.0 (moderate) | 66.0 ± 6.69 | 62.25 ± 5.17 | -3.75 | -0.52 (moderate)  (-1.22, 0.17) | 0.30  (0.21, 0.62) |  |
| Depression | 3.5 (small) | 57.22 ± 3.76 | 59.56 ± 2.49 | -2.34 | -0.58 (moderate)  (-1.25, 0.09) | 0.26  (0.12, 0.50) |  |

Desired effect. Undesired effect. Trivial effect/Overlap. A = non-intervention phase. B = intervention phase. NAP = non-overlap of all pairs. SD = standard deviation. WC-SMD – within case standardized mean difference. 95%CI = 95% confidence interval. ∆ = mean difference.
